# Prevalence of Bacterial Contamination of Casting Material in a Pediatric Population

**DOI:** 10.1155/2020/4717385

**Published:** 2020-01-21

**Authors:** Brett Walker, Chad Amato, Olena Palyvoda, Sharada Vangipuram, Martin Weaver, Zain Sayeed, Muhammad Talha Padela, Walid K. Yassir

**Affiliations:** ^1^Department of Orthopedics, DMC Children's Hospital of Michigan, Detroit, MI 48201, USA; ^2^Department of Chemistry, Wayne State University, Detroit, MI 48201, USA; ^3^Wayne State University School of Medicine, Detroit, MI 48201, USA; ^4^Resident Research Partnership, Detroit, MI 48201, USA; ^5^FAJR Scientific, Detroit, MI 48201, USA

## Abstract

Surgical site infection is a relatively common and devastating complication following pediatric orthopedic surgery. Many infections have been determined to be the result of settled airborne particles on surgical equipment and the sterile field. Fiberglass casts are commonly used orthopedic fixation devices before and after surgery; however, fiberglass casting material is expelled during the removal process and represents an uninvestigated area for the possibility of cast saw dust as a source of airborne bacterial contamination in an operating room setting. This study evaluates the prevalence and distribution of microbiota on 90 pediatric casts by collecting and culturing fiberglass cast material from 90 pediatric casts. Bacterial identification was performed using a Bruker Biotyper Matrix-Assisted Laser Desorption/Ionization Time-of-Flight Mass Spectrometry device. 81 out of 90 casts (90%) showed evidence of microbial contamination. Isolated species were very diverse and ranged from normal skin flora to opportunistic pathogens. The 5 most commonly isolated organisms were *Acinetobacter pittii, Enterobacter cloacae, Micrococcus luteus, Staphylococcus epidermidis,* and *Staphylococcus hominis. *Further investigation is required to determine if casting material is truly a cause of surgical site infection.

## 1. Introduction

Surgical site infections (SSI) are an incredibly costly and damaging complication following pediatric orthopedic surgical procedures. SSIs are the most common cause of nosocomial infections with 300,000–500,000 occurring annually in the United States alone [1]. Infection after pediatric orthopaedic surgery is an understudied topic. Studies suggest that the rate of infection in children is lower than the rate in adults; however, infections are still a relatively common complication following orthopedic surgery affecting up to 2.9% of children after fracture fixation with hardware [2]. In addition to decreasing patient's wellbeing and introducing significant morbidity, SSIs have been demonstrated to increase average length of stay from 5 to 13 days, increase cost of stay by thousands of dollars and significantly increase risk of post-procedural mortality [3, 4]. Given that SSIs are preventable complications, there has been significant research investigating how to minimize their occurence. Current models classify SSIs as either being due to patient-related factors, including comorbidities such as diabetes, nicotine use, COPD, age, sex and BMI or secondary to intra-operative behaviors such as sterilization and hand-washing procedure and surgical team behavior. It has been demonstrated that intraoperative events, behaviour, and protocol have a significantly greater effect on infection than patient-related measures [5]. Advances in air filtration, pre-surgical scrub, and surgical site preparation have all played a significant role in decreasing surgical site infections [6]. Despite this progress, studies have demonstrated that the vast majority of surgical site contamination in orthopedic surgical procedures is still due to airborne particles settling in the wound or on instrumentation [7]. Minimizing this source of surgical site contamination should be a high priority for improving patient surgical outcomes as well as decreasing risk and cost of procedures.

The use of fiberglass casts is incredibly common in pediatric orthopedic practices due to the lightweight and low profile of casts, relative ease of application and removal, and high patient compliance while still providing excellent fracture immobilization [8]. While casts are an effective solution for fracture immobilization and removal is relatively simple and rapid prior to procedures in the operating room, the removal of fiberglass casts generates a significant amount of airborne debris which could act as a possible source of airborne contamination in pediatric orthopedic surgery. There has been little investigation exploring the bacterial colonization of casts as a possible source of surgical site infections in a pediatric population. This study seeks to identify bacterial contamination, common colonizers, and possible bacterial pathogens of casts in a pediatric population. We desire that the results of this study be used to direct further research to evaluate whether casting material poses a credible risk for surgical site infection and for clinicians to consider whether cast removal should be performed prior to the patient's entrance into the operating suite.

## 2. Materials and Methods

Prior to the initiation of this study and collection of any data, this study was approved by Wayne State University's institutional review board. Between June and July of 2015, ninety sequential fiberglass casts were removed in a controlled fashion at an academic pediatric orthopedic clinic for analysis. The location of the cast's application (upper or lower extremity), type of cast applied (long, short, or spica) and length of time cast was in place, was recorded using a data collection tool. Obviously and severely water damaged casts were excluded from the study so as to standardize and normalize the sample collection as much as possible. Cast removal and sample collection was performed in a standardized, sequential fashion by one gloved investigator (CA) using a Stryker (Kalamazoo, MI) oscillating cast saw, cast spreader, and bandage scissors. The blade from the saw and an unopened roll of fiberglass cast type were cultured before the study to ensure they were not a source of bacterial contamination. One roll of fiberglass material was determined to be appropriate for testing given the consistent nature of packaging, handling, and sterilization by the manufacturer. Immediately after cast removal, the investigator collected samples of fiberglass dust freshly cut from the external ½ thickness of the cast to ensure cotton padding was excluded from the sample. The removed fiberglass casting material was collected with a sterile tongue depressor and placed into sterile test tubes which were immediately sealed. These tubes contained no bacterial medium; only dry samples were collected. At the end of each day (a maximum of 3 hours after collection), the examiner personally took all samples to the department of microbiology for analysis. Collected samples were suspended in 1 mL of sterile normal saline and plated on labelled blood agar dishes. Cultures were incubated at 35 degrees Celsius for 48 hours and were turned 3 times to ensure adequate coverage. 81 of the 90 cast cultures taken had microbial growth following incubation; from these colonies, 1014 random bacterial colony samples were isolated and evaluated.

Colony analysis was performed by the Bruker Biotyper Matrix-Assisted Laser Desorption/Ionization Time-of-Flight Mass Spectrometry (MALDI-TOF MS) System to calculate mass and distribution of ribosomal proteins. The resultant mass spectrum is species specific and allowed examiners to quickly identify the isolated bacterial species from a large database. For our study, we utilized the BRUKER database with 5627 entries from 7 Archaea, 4970 Bacterial and 650 Eukaryotic species. Results were recorded from the analysis and tabulated. Samples which were identified to the genus and species level were included while those who were unable to be identified to this level were excluded.

## 3. Results

Ninety sequential, fiberglass casts were examined in the study of which 33 were short arm; 26 were long arm; 17 were short leg; 10 were long leg; and 4 were hip Spica casts. 81 out of the 90 casts (90%) which were cultured were confirmed to have bacterial colonization. Nine casts (10%) were objectively demonstrated to be free of contamination. Control samples from freshly opened casting material and the Stryker saw blade were found to be free of contamination as well. Bacterial classification was performed and in 77 casts, bacteria were identified to the species level (85.5%), in 4 casts bacteria were identified to the genus level (4.5%). Classification revealed sixty-one unique species of bacteria that had colonized casting material during the study. A complete list of bacterial isolates is listed in (Table 1). Analysis of bacterial isolates revealed many samples were normal skin flora; however, some isolates were fairly atypical skin microbiota. The most prevalent bacteria observed were *Staphylococcus Hominis* (48.15%), *Staphylococcus Epidermidis* (29.63%), *Micrococcus Luteus* (25.93%), *Enterobacter Cloacae* (16.05%), *Acinetobacter Baumannii* (14.81%), and *Acinetobacter Pittii* (12.35%). Each of these species were present in over 10% of samples collected.

## 4. Discussion and Conclusions

Despite advances in sterile technique, antimicrobial prophylaxis, and post-operative optimization of patients, surgical site infections continue to be the most common cause of nosocomial infections and continue to be a significant cause of morbidity and mortality post operatively as well as decreasing patient satisfaction and dramatically increasing cost of procedure and hospital stays. Many centers strictly limit traffic into and out of the operative suite and do not allow outside items to be brought into the room as they may be sources of contamination. Most operating rooms now have laminar air flow and antimicrobial filters to attempt to minimize airborne pathogens and it has been shown, implementing these measures leads to a significant decrease in the incidence of surgical site infection during implant surgery [9]. All operating rooms have extensive processes in place to sterilize equipment or ensure disposable equipment is sterile and available. Many medical centers now ensure operating room staff must undergo specialized training to ensure scrub technique is sufficient and equipment and the sterile field remains undisturbed as per World Health Organization standards. Despite these improvements, contamination of the surgical field continues to be problematic and surgical site infection is still relatively prevalent. This present study describes an identifiable and likely avoidable source of bacterial contamination in orthopedic surgery. We are unaware of any other studies that have investigated the microbial load or diversity of fiberglass casts nor that have investigated the possibility that operative suite cast removal could present a preventable cause of surgical site infections in pediatric orthopedic surgery. In the present study, 90% of fiberglass casts examined contained polymicrobial bacterial contamination. Only 16.4% of samples were considered to be normal skin flora, while other bacteria identified have been implicated in sepsis, meningitis, pneumonia, multidrug resistance and surgical site infections [10, 11]. Many of the samples contained bacteria that are resistant to normal cleaning procedures, with one being resistant to radiation exposure (*A*. *radioresistens*). Quite a few of the isolated bacterial species are highly pathogenic and many have been documented to be opportunistic pathogens. In one study, *A*. *baumannii* was responsible for 19.1% of ventilator associated pneumonia cases [12], while *Citrobacterkoseri* is responsible for devastating meningitis and brain abscesses in at risk neonates [13]. *A. Pitti *has recently found to be an increasingly common organism isolated in patients with bacteremia [14]. *Enterobacter *species were commonly isolated from cast specimens and some studies have implicated this genus in up to 7% of nosocomial infections in the United States [15]. More serious infections ranging from bacterial meningitis and cerebral abscesses to necrotizing fasciitis have been reported to be a result of *Enterobacter Cloacae* as well [16, 17]. *Micrococcus luteus *has been reported in the development of cases of endocarditis and septic arthritis [18, 19]. Recent literature agrees that *Staphylococcus epidermidis* is one of the most frequent pathogens underlying the development of bacteremia and suggests that bacterial sepsis from *S. epidermidis *may be more severe than *S. Aureus* as *S. epidermidis *may produce more severely pro-inflammatory toxins [20]. Similarly *S. Hominis *and *S. haemolyticus* have been identified as opportunistic pathogens involved in infection of newborns and as nosocomial pathogens of bacteremia and sepsis with relatively similar and high mortality [21]. Other less commonly encountered organisms were also investigated and found to have been implicated and reported to cause many nosocomial pathologies. It is important to note that even normal skin flora can also be pathogenic when introduced into a fresh surgical wound [22].

While this study demonstrates that casting material is contaminated with many bacterial organisms including normal skin flora and possible pathogenic species; there are some limitations to this research. Mainly, that this data does not demonstrate the fiberglass cast dust contaminates the surgical field and can actually be a cause of airborne contamination of the surgical field. Surgical site infection is a serious problem and minimizing risks should be an important priority in the operating room; further investigation should definitely prioritize the evaluation of whether or not casting material has infected surgical equipment or surgical team members and resulted in the development of infection. If further study is able to corroborate that contaminated casting material can and does contaminate the surgical field, it may be wise to end the practice of cast removal in the operating suite to further decrease the risk of surgical site infection. Another important point to consider is that there are many available options for fracture and post operative surgical immobilization that could eliminate the risk of microbially contaminated fiberglass dust. Braces and splints do not require removal with a cast saw which could make them a superior option for fracture/orthopedic mobilization without increased risk for surgical site infection. Unfortunately, there is little recent quality clinical evidence comparing the efficacy between fracture and orthopedic immobilization options, especially in a pediatric population. Some studies suggest that bracing or taping may be non-inferior to casting for certain injuries including patellar dislocation; however, these studies are underpowered [23]. For ankle fractures, recent data suggests that casting may be more effective than bracing to optimize partial weightbearing; however a meta-analysis evaluating bracing and casting following achilles tendon injury favored bracing after surgery [24, 25]. Another study comparing ankle fractures and immobilization options found increased early range of motion and subjective scores in braced patients with stable non-surgical ankle fractures but increased rates of complications especially wound infection and dehiscence in patients following unstable surgically fixated ankle fractures [26]. Biomechanical data suggests that there is no significant difference between cadaveric distal radius fractures braced with heat molded splints and fiberglass casts [26]. Overall, the data comparing immobilization options is not consistent recommending a specific technique. It is also important to consider that in a pediatric population, casting is generally preferred as it prevents patients from removing their own fixation device which drastically improves compliance.

Strengths of our study include a large sample size and a consistent, reproducible technique of cast removal and bacterial isolation and culture. The use of the MALDI Biotyper allowed the examiners to quickly identify bacteria to the species level for evaluation of the depth of microflora present and for possible pathogens. Bacterial colonies examined were selected at random during this study, however, many colonies were not sampled. Changing which colonies were sampled could potentially have an effect on the prevalence of particular bacteria in data and what species could be represented. It is also possible that transporting only dry sample could have excluded less hardy organisms from the study and could also explain the very low rate of anaerobes that were isolated. We believe, however, because such a large percentage of samples were found to be contaminated, this would not affect the implications of the study.

Important conclusions of this study include that there is a fairly large microbial load and diversity in fiberglass casting material including many opportunistic pathogens. Given that fiberglass cast removal commonly occurs in the operative suite before internal fixation and that airborne contaminants have been shown to be the main cause of surgical site infections in pediatric orthopedic surgery, it may be prudent to consider performing cast removal outside of the operative suite; however, further research is needed to truly verify whether casting material can definitively lead to surgical site infection.

## Figures and Tables

**Table 1:** Identification of bacteria within casts.

Bacteria	# of samples	% of samples
*Staphylococcus hominis*	39	48.15
*Staphylococcus epidermidis*	24	29.63
*Micrococcus luteus*	21	25.93
*Enterobacter cloacae*	13	16.05
*Acinetobacter baumannii*	12	14.81
*Acinetobacter pittii*	10	12.35
*Cornyebacterium afermentans*	5	6.17
*Staphylococcus xylosus*	5	6.17
*Bacillus megaterium*	4	4.94
*Pseudomonas monteilii*	4	4.94
*Arthrobacter creatinolyticus*	3	3.70
*Enterobacter hormaechei*	3	3.70
*Pseudomonas aeruginosa*	3	3.70
*Staphylococcus capitis*	3	3.70
*Staphylococcus pettenkoferi*	3	3.70
*Staphylococcus sciuri*	3	3.70
*Acinetobacter nosocomialis*	2	2.47
*Bacillus cereus*	2	2.47
*Bacillus safensis*	2	2.47
*Citrobacter koseri*	2	2.47
*Enterobacter asburiae*	2	2.47
*Erwinia *sp	2	2.47
*Klebsiella pneumoniae*	2	2.47
*Leclercia adecarboxylata*	2	2.47
*Micobacterium arborescens*	2	2.47
*Micrococcus lylae*	2	2.47
*Staphylococcus saprophyticus*	2	2.47
*Stentrophomonas maltophilia*	2	2.47
*Wautersiella falsenii*	2	2.47
*Acinetobacter johnsonii*	1	1.23
*Acinetobacter iwoffii*	1	1.23
*Acinetobacter junii*	1	1.23
*Acinetobacter radioresistens*	1	1.23
*Acinetobacter schindleri*	1	1.23
*Alcaligenes faecalis*	1	1.23
*Bacillus indicus*	1	1.23
*Bacillus pumilus*	1	1.23
*Brevundimonas diminunta*	1	1.23
*Cornyebacterium jeikeium*	1	1.23
*Cornyebacterium minutussumum*	1	1.23
*Cornebacterium multifaciens*	1	1.23
*Curtobacterium albidum*	1	1.23
*Enterobacter cowanii*	1	1.23
*Enterobacter ludwigii*	1	1.23
*Enterococcus casseliflavus*	1	1.23
*Kocuria rhizophila*	1	1.23
*Microbacterium testaceum*	1	1.23
*Ochrobactrum intermedium*	1	1.23
*Ooligella urethralis*	1	1.23
*Pantoea dispersa*	1	1.23
*Pseudomonas fluva*	1	1.23
*Pseudomonas oryzihabitans*	1	1.23
*Pseudomonas putida*	1	1.23
*Pseudomonas equi*	1	1.23
*Rhodococcus equi*	1	1.23
*Shewanella Baltica*	1	1.23
*Staphylococcus cohnii*	1	1.23
*Staphylococcus condimenti*	1	1.23
*Staphylococcus equrum*	1	1.23
*Staphylococcus haemolyticus*	1	1.23
*Staphylococcus simulans*	1	1.23

## Data Availability

The data used to support the findings of this study are available from the corresponding author upon request.
